# One-year outcome of low dose laser cyclophotocoagulation for capsular tension ring-induced malignant glaucoma: A case report

**DOI:** 10.1097/MD.0000000000018836

**Published:** 2020-02-07

**Authors:** Haishuang Lin, Guangming Zhou, Shaodan Zhang, Fang Huang, Yuanbo Liang

**Affiliations:** a The Eye Hospital of Wenzhou Medical University, Zhejiang Eye Hospital; b Glaucoma Institute of Wenzhou Medical University, Wenzhou, Zhejiang Province, China.

**Keywords:** capsular tension ring, low dose laser cyclophotocoagulation, malignant glaucoma

## Abstract

**Rationale:**

Malignant glaucoma is a refractory glaucoma which often relentlessly worsened despite conventional therapy. Ultrasonographic biomicroscopy always cannot show the ciliary-block of malignant glaucoma. We report a case of capsular tension ring induced ciliary-block and successfully treated by low dose laser cyclophotocoagulation, with 1-year follow-up.

**Patient concerns:**

A 75-year-old woman was referred for glaucoma with a history of cataract and lens zonular laxity, and surgery with combined phacoemulsification and capsular tension ring implantation. She subsequently underwent trabeculectomy for uncontrolled intraocular pressure on maximal medical therapy. One day later, the patient presented as shallow anterior chamber of Shaffer grade 1 and an elevated intraocular pressure of 51.0 mmHg in the right eye.

**Diagnosis:**

Ciliary block caused by capsular tension ring and malignant glaucoma was observed.

**Interventions:**

Low dose laser cyclophotocoagulation was performed under retrobulbar anesthesia.

**Outcomes:**

One day later, the patient's intraocular pressure decreased to 14.3 mmHg on topical atropine 1% and 2 classes of intraocular pressure lowering medications. The patient discontinued topical atropine and intraocular pressure lowering medications 4 months postoperatively and her condition had remained stable for 1 year without any medications. The patient had a satisfactory recovery benefited from the low dose laser cyclophotocoagulation.

**Lessons:**

Low dose laser cyclophotocoagulation in this challenging case of capsular tension ring-induced malignant glaucoma provided an effective and fast recovery of anterior chamber depth over a 1-year period.

## 1 Introduction

Malignant glaucoma (MG), first reported by Von Graefe in 1869, presents as a clinical challenge to cure because it relentlessly worsened despite conventional therapy.^[1]^ The prevalence of MG among primary angle-closure glaucoma (PACG) patients who accepted incisional surgery ranging from 0.6% to 4%.^[2]^ It is usually characterized by a flattening of the anterior chamber (AC) and elevated intraocular pressure (IOP) in the absence of a pupillary block. However, ultrasonographic biomicroscopy (UBM) always cannot show the ciliary-block of malignant glaucoma. Currently, the main treatment for MG includes medication, laser capsulohyaloidotomy, or surgical disruption of zonulo-hyaloido-vitrectomy (and phacoemulsification if phakic), with the goal of deepening AC and lowering IOP.

Laser cyclophotocoagulation (CP) was widespread used for refractory glaucoma.^[3]^ The preliminary use of this technique for MG was first described by Carassa et al^[4]^ in 1999, then by Stumpf et al^[5]^ in 2008. They recommend it as an early treatment before zonulo-hyaloido-vitrectomy, given the success of treatment for MG cases. Based on the clinic experiences, the application of pulses and quadrants of CP was set for low dose so as to reduce complications and prevent future recurrences.

This article reports the successful use of low dose laser cyclophotocoagulation (LDCP) in a MG patient after combined phacoemulsification and capsular tension ring (CTR) implantation whose ciliary block between CTR and ciliary body was clearly shown in UBM. Mechanisms of how capsular tension ring-induced malignant glaucoma (CTR-induced MG) developed and how LDCP relieve the ciliary-block in this patient were also postulated.

## 2 Case presentation

A 75-year-old woman was referred for further treatment for uncontrolled intraocular hypertension following trabeculectomy. The patient had a past medical history significant for hypertension. Her family history was negative for glaucoma. She had a past ocular history significant for cataract in both eyes and lens zonular laxity in right eye. She underwent right phacoemulsification with intraocular lens (IOL) and CTR (Holland, OPHTEC B.V., 13.0 mm in noncompressed diameters) implantation in November of 2017. Following surgery, the best corrected visual acuity (BCVA) was 20/22 and IOP was 11.1 mmHg in the right eye. The slit-lamp examination showed that anterior segment examination was unremarkable. Twelve days after cataract surgery, elevated IOP of 41.2 mmHg in the right eye was noted and she underwent laser peripheral iridotomy and was placed on medical therapy for intraocular hypertension.

**Figure 1:**
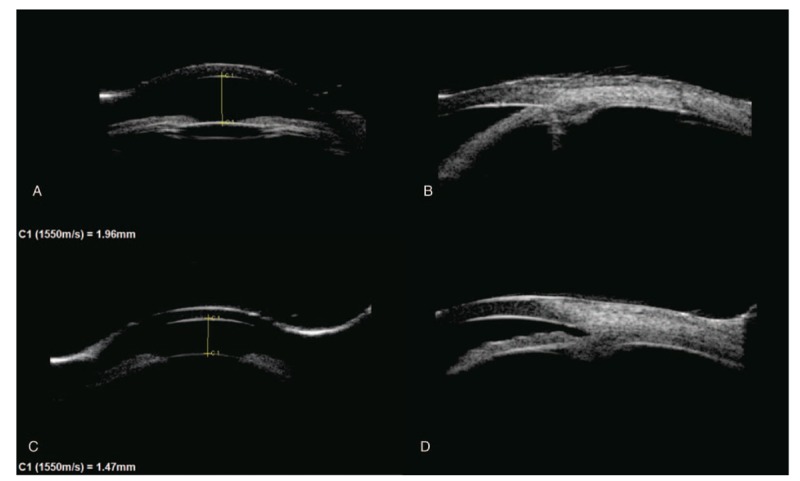
A and B, UBM showed shallowing of the AC and angle closure in the right eye. C and D, UBM showed shallowing of the AC and angle open in the left eye. B and D showed inferior anterior chamber angle. AC = anterior chamber, UBM = ultrasonographic biomicroscopy.

Several months with treatment of maximal medical therapy, right IOP remained uncontrolled. She presented with complaints of pain on March 20, 2018. On examination, visual acuity (VA) was 20/200 in the right eye and 20/33 in the left eye. IOPs were 39.5 mmHg in the right and 15.6 mmHg in the left eyes on fixed combination brimonidine 0.2%/timolol 0.5%/brinzolamide 1% bid and bimatoprost 0.03% qn in the right eye, respectively. Slit-lamp examination revealed peripheral AC <1/4 corneal thickness in both eyes, patent peripheral iris incisions (PIs) and narrow closed angles in the right eye, narrow open angles in the left eye, and cup to disc ratios of 0.3 in both eyes. Posterior chamber IOL was in good position in the right eye and early senile cataract was present in the left eye. Axial length is 20.72 mm in the right eye and 20.69 mm in the left eye. Len-star showed that white to white is 11.6 mm in the right eye and 10.4 mm in the left eye. UBM showed shallowing of the AC in both eyes and angle closure in the right eye (Fig. 1A–D). A diagnosis of secondary angle-closure glaucoma was made. Given her pain and uncontrolled IOP, she underwent trabeculectomy on March 21, 2018.

**Figure 2:**
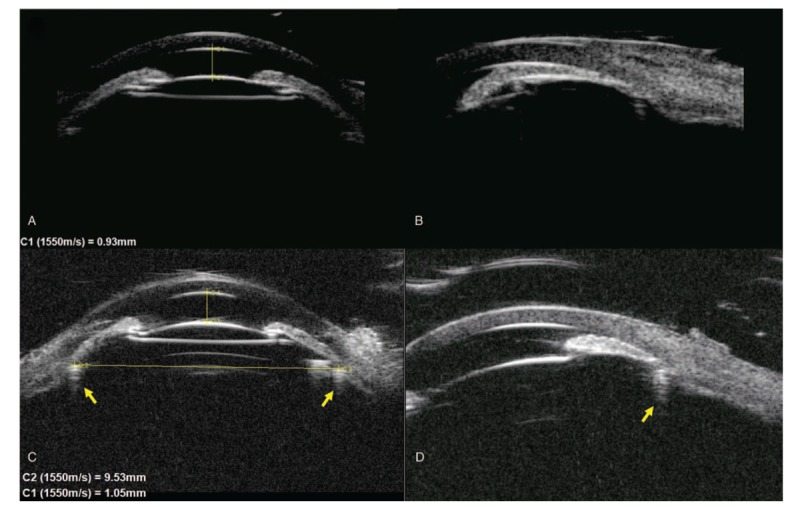
A and B, UBM showed ciliary block on day 1 after trabeculectomy. C and D, UBM showed that ciliary sulcus diameter (horizontal yellow line) had smaller size than CTR and the ACD deepened slightly after maximum topical medical therapy. B and D showed inferior anterior chamber angle. Yellow arrow showed the acoustic shadow of CTR. ACD = anterior chamber depth, CTR = capsular tension ring, UBM = ultrasonographic biomicroscopy.

The surgery was uneventful except the presenting of shallow AC of Shaffer grade 1 and relieved by tropicamide. However, the IOP quickly increased to 43.5 mmHg during the first hour after the operation. The slit-lamp examination was significant for 2+ corneal edema. In an effort to reduce her IOP, the filtering bleb was massaged. On postoperative day 1, the patient presented as shallow AC of Shaffer grade 1, uncorrected VA was counting fingers and an elevated IOP of 51.0 mmHg in the right eye. UBM showed ciliary block between CTR and ciliary body (Fig. 2A and B). Since there were no signs of choroidal detachment or suprachoroidal hemorrhage, a diagnosis of MG was made. Subsequently, the patient underwent posterior capsulotomy by Nd: YAG and accepted oral acetazolamide and maximum topical medical therapy (atropine 1%, prostaglandin analog, timolol, bromonidine, brinzolamide) for 4 days. However, AC remained shallow and IOP was uncontrolled. UBM was done again on March 20, 2018 and high attenuation shadow of CTR was noticed (Fig. 2C and D).

Given presence of unacceptable IOP, it was considered to surgically manage her ciliary block glaucoma. However, whether to remove the CTR was a problem. It seemed that removal of CTR was the most effective way to relive ciliary block as well as the most difficult procedure. Given our personal successful experience with the CP in patients with refractory glaucoma in shallow AC, we decided on performing LDCP first. Pars plana vitrectomy and CTR removal was also prepared in case of the failure of LDCP.

The Ethics committee of the eye hospital of Wenzhou Medical University approved the conduct of LDCP as a new therapy for MG (YX2018-015). An informed consent was obtained from the patient.

LDCP was performed under retrobulbar anesthesia with OcuLight SLx semiconductor diode 810 nm laser and the contact G-probe on March 27, 2018. The ciliary body was identified by transillumination and the contact G-probe was placed at the anterior aspect of the ciliary body which approximately 1.8 mm posterior to the limbus. The treatment consisted of 15 applications of 2.0 W energy applied for 2 seconds over 6 to 9 o’clock (avoiding the 9 o’clock position, 90° total) of the ciliary body. Upon termination of the procedure, atropine and dexamethasone ointments were placed on the eye to reduce the postoperative inflammation.

**Figure 3:**
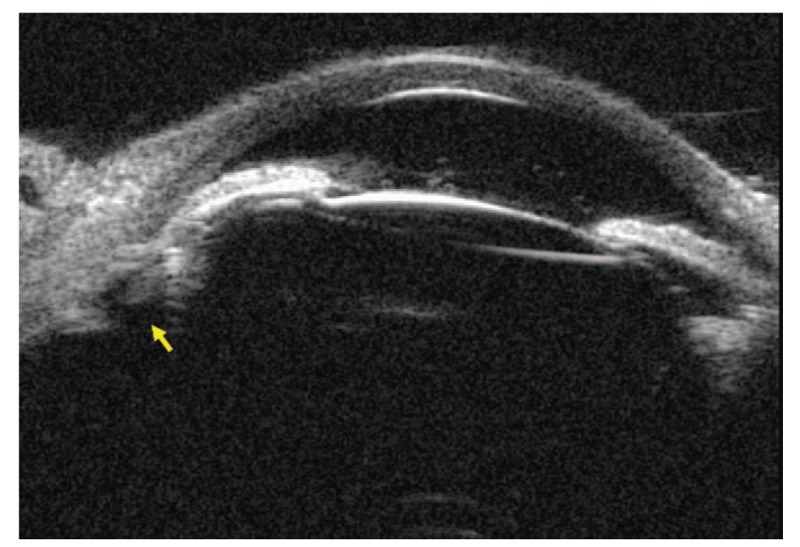
UBM showed the relief of inferior ciliary block on day 2 after LDCP. Yellow arrow may be a patent communication from the posterior space of the lens to the posterior chamber caused by LDCP. LDCP = low dose laser cyclophotocoagulation, UBM = ultrasonographic biomicroscopy.

**Figure 4:**
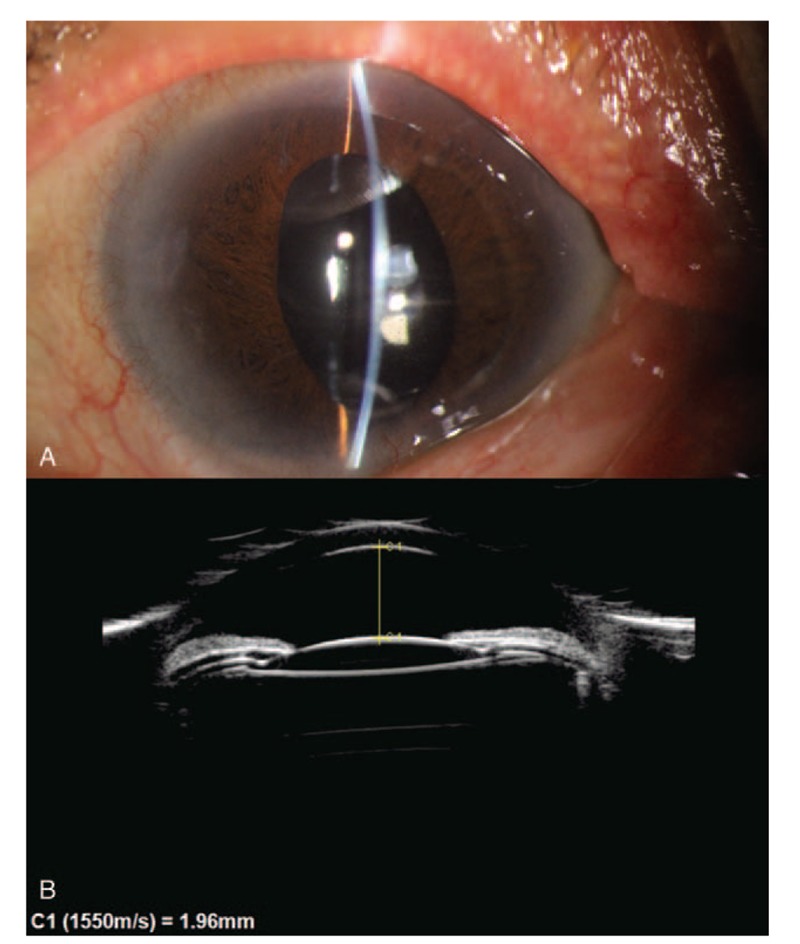
A, Slit-lamp examination and UBM showed the depth of central AC in the right eye during 1 year of follow-up. B, The ACD sustained 1.93 mm. AC = anterior chamber, ACD = anterior chamber depth, UBM = ultrasonographic biomicroscopy.

Three hours after LDCP treatment, IOP in the right eye decreased to 24.9 mmHg with the ease of corneal edema and improvement of transparency, and uncorrected VA was 20/250 in the right eye. She was treated with atropine, dexamethasone ointments, timolol, and brinzolamide. On postoperative day 2, the IOP was 22.7 mmHg with a clear cornea and 20/167 vision, and bimatoprost 0.03% qn was added. UBM showed the relief of inferior ciliary block (Fig. 3) and a suspect communication from the posterior space of the lens to the posterior chamber. On the day 7 visit, the IOP was 15.9 mmHg on 5 medications and 3 medications was reduced. The patient was seen at the 1-month postoperative visit with an IOP of 17.0 mmHg on atropine bid/bimatoprost 0.03% qn, and her uncorrected visual acuity (UCVA) in the right eye improved to 20/100. UBM confirmed a well-relief of ciliary block with an anterior chamber depth (ACD) of 1.70 mm. At 3 months, IOP measured 17.5 mmHg on atropine bid/bimatoprost 0.03% qn. The patient discontinued topical atropine and bimatoprost 4 months postoperatively. During her 6 months of follow-up, her IOP was 18.9 mmHg and ACD was 2.09 mm without any medication. At her last visit on March 29, 2019, VA remained 20/167, IOP measured 16.5 mmHg in the right eye. Slit-lamp examination and UBM showed the depth of central AC in the right eye (Fig. 4A and B). There was no significant uveitis and other complications observed during 1-year follow-up.

## 3 Discussion

LDCP in this special patient effectively reduced IOP and deepen central anterior AC over a 1-year period. Patients with MG are typically treated with zonulo-hyaloido-vitrectomy when uncontrolled IOP and shallow AC are encountered. Previous studies have reported that the success rates of zonulo-hyaloido-vitrectomy ranged from 34.0% to 60.0% in treating for MG.^[6,7]^ However, our patient had previously been implanted with CTR which contacted with ciliary closely, leading to ciliary-block. CTR-induced MG has been infrequently reported in the literature. Given the difficulty of CTR removal and limited success rates of zonulo-hyaloido-vitrectomy, LDCP was considered.

CP is a clinical approach that aims to reduce IOP by ablating the ciliary body to reduce the production of aqueous humor, and thus lowering the IOP. CP is traditionally used for the treatment of refractory glaucoma.^[2]^ For malignant glaucoma, CP is conventionally reserved for cases with poor visual prognosis or failed of Nd: YAG laser hyaloidotomy and vitreolysis.^[4]^ Stumpf et al^[5]^ also reported 5 patients with MG successfully treated with CP and suggests that CP is an easy and effective treatment for some cases of MG.

However, there is no standard guidance for clinicians who want to perform CP as a therapeutic intervention for refractory glaucoma. And the substantial risk for vision loss, hypotony, and phthisis, as well as typically less predictable outcome^[8–10]^ disturbed clinicians for a long time. Recently, Bendel and Patterson^[11]^ pointed out that CP can be considered earlier for treating refractory glaucoma and can be used in a variety of glaucoma types. And CP has been used as a primary surgery in eyes with relatively good visual potential.^[12–15]^ The rates of serious complications seem to decrease, which may be related to the lower energy settings and less severe forms of glaucoma than in prior studies.^[8,16]^

The major etiologic factor of MG is thought to be the ciliary block resulting from forward movement of the iris-lens diaphragm.^[17]^ In our case, the patient had a typical presentation of CTR, which contacted with ciliary closely and caused MG directly. Meanwhile, the patient also had risk factors include zonular laxity, short axial length,^[18]^ and history of trabeculectomy. Herschler^[19]^ observed that a relief of the ciliary block occurred after laser shrinkage of the ciliary in 6 MG patients. In order to reduce side effects and make use of possible effect of shrinking ciliary, we modified the energy settings and pulses of CP which we called LDCP. The new parameter included 15 pulses over 90° to 120°, 2.0 W energy, 2 seconds duration. And LDCP was no more a destructive procedure for its limited range. Thus, LDCP was a viable option for this patient.

This is a single case of a unique patient with CTR induced MG that successfully treated by LDCP. Apparent ciliary block resulted from the contact of CTR and ciliary body. It emphasized the potential risk of the CTR. The zonular space can be compressed by the IOL and CTR, leading to the coincident of capsular bag diameter and the ciliary ring diameter.^[20]^ We hypothesize that LDCP is likely to establish a patent communication from the posterior space of the lens to the posterior chamber which blocked by CTR and ciliary body (Fig. 3) and separating the area of ciliolenticular touch. With the breaking of the cycle of aqueous misdirection, the anatomic relationships between anterior segment and posterior segment could be restored and the “malignant” process could be broken. The advantages of CP include lower to cost, less difficulty to operate, and fewer chances of postoperative infection compared with zonulo-hyaloido-vitrectomy. Importantly, if this approach is not successful it does not complicate a subsequent surgical approach.^[5]^

In our patient, a significant and sustained ACD deepen was achieved. Given this experience and possible mechanism of MG, LDCP may be considered prior to surgical intervention in MG case where medical treatment has been insufficient. More experience is needed for the efficacy and safety of LDCP, but LDCP seems to be promising.

## Author contributions

**Data curation:** Haishuang Lin, Guangming Zhou, Shaodan Zhang, Fang Huang.

**Formal analysis:** Haishuang Lin, Guangming Zhou, Shaodan Zhang, Yuan Bo Liang.

**Investigation:** Haishuang Lin, Guangming Zhou, Shaodan Zhang.

**Methodology:** Haishuang Lin, Guangming Zhou, Shaodan Zhang, Yuan Bo Liang.

**Resources:** Yuan Bo Liang.

**Supervision:** Fang Huang, Yuan Bo Liang.

**Validation:** Fang Huang, Yuan Bo Liang.

**Writing – original draft:** Haishuang Lin.

**Writing – review & editing:** Haishuang Lin, Guangming Zhou, Shaodan Zhang, Fang Huang, Yuan Bo Liang.
